# Lactate Dehydrogenase/Albumin To-Urea Ratio: A Novel Prognostic Maker for Fatal Clinical Complications in Patients with COVID-19 Infection

**DOI:** 10.3390/jcm12010019

**Published:** 2022-12-20

**Authors:** Hala Shokr, Mandeep Kaur Marwah, Hisam Siddiqi, Farooq Wandroo, Lissette Sanchez-Aranguren, Shakil Ahmad, Keqing Wang, Sukhjinder Marwah

**Affiliations:** 1 Pharmacy Division, School of Health Sciences, Faculty of Biology, Medicine and Health, The University of Manchester, Manchester M13 9PL, UK; 2 Aston Medical School, College of Health and Life Sciences, Aston University, Birmingham B4 7ET, UK; 3 Department of Haematology, Sandwell and West, Birmingham Hospitals NHS Trust, West Bromwich B71 4HJ, UK

**Keywords:** COVID-19, SARS-CoV, prognosis, biomarkers, lactate dehydrogenase, albumin ratios

## Abstract

Objective: To investigate lactate dehydrogenase/Albumin to-urea (LAU) ratio as a potential predictor for COVID-19-induced fatal clinical complications in hospitalized patients. Methods: This is a retrospective study involving blood analyses from 1139 hospitalised COVID-19 infection survivors and 349 deceased cases post-COVID-19 infection. Laboratory tests included complete blood picture, inflammatory markers, and routine organ function tests. Results: The non-survivor group showed lower haemoglobin (*p* < 0.001), platelet (*p* < 0.0001) and higher mean corpuscular volume, neutrophil count, neutrophil/lymphocytes ratio (NLR), and LAU (*p* < 0.001, *p* < 0.0013, *p* < 0.001, *p* < 0.0126) than the patients who survived the infection. The non-survivors also exhibited higher markers for infection-related clinical complications, such as international normalized ratio (INR), D-dimer, urea, total bilirubin, alkaline phosphatase (ALK), creatinine, c-reactive protein (CRP), and serum ferritin levels (all *p* < 0.05). In addition, LAU ratio was positively correlated with infection prognostic parameters including INR (r = 0.171), D-dimer (r = 0.176), serum urea (r = 0.424), total bilirubin (r = 0.107), ALK (r = 0.115), creatinine (r = 0.365), CRP (r = 0.268), ferritin (r = 0.385) and negatively correlated with serum albumin (r = −0.114) (*p* ≤ 0.05). LAU ratio had an area under receiver operating characteristic of 0.67 compared to 0.60 with NLR. Conclusion: Patients with a high LAU ratio are at increased risk of mortality due to COVID-19 infection. Therefore, early assessment of this parameter, intensive intervention and close monitoring could improve their prognosis.

## 1. Introduction

Coronaviruses consist of a range of viruses that cause mild to severe upper respiratory tract infection [1]. In the past two decades, three beta-coronaviruses have evolved into several pandemics: SARS coronavirus (SARS-CoV), which caused the severe acute respiratory syndrome (SARS) pandemic in 2002 [2]; MERS coronavirus (MERS-CoV), which emerged in 2012 and caused Middle East respiratory syndrome (MERS) pandemic [3]; and SARS-CoV-2, which shares 79% sequence identity with SARS-CoV and was behind the COVID-19 pandemic in 2019 [4]. Although patients infected with these viruses usually present with easily managed flu-like symptoms, the 2019 variant was found to be more contagious and also associated with higher in-hospital mortality rates [5].

According to the official UK government website for data and insights on COVID-19, more than 3000 patients are newly diagnosed with the virus every week with a mortality rate above 14% (approximately 433 patients) due to respiratory complications [6].

Similarly, to date, approximately 30% of cases were observed to develop acute respiratory distress syndrome (ARDS) requiring mechanical ventilation, with the additional risk of progression to viral coagulopathy, respiratory failure, shock and multiorgan failure (MOF) [7]. Additionally, even patients with normal or mild symptoms can, sometimes, deteriorate rapidly to fatal complications, making routine clinical assessment an unreliable predictor for the prognosis of such patients [8].

Over the past three years, aberrant inflammation and proinflammatory cytokine storm were recognized as the main events implicated in the pathophysiology of COVID-19-related clinical complications and mortality. Consequently, scientists considered inflammatory mediators as possible biomarkers of multiorgan failure (MOF) in these patients [5]. Among them, neutrophilia, lymphopenia, and thrombocytopenia were found to be correlated with disease severity in many populations [9,10]. Additionally, acute phase reactants, such as lactate dehydrogenase (LDH), ferritin (FER), C-reactive protein (CRP) and D-dimer (DD) were found to be prominent in the MOF phase of the infection [11]. Furthermore, some haematological ratios, e.g., neutrophil-to-lymphocyte ratio (NLR) and platelet-to-lymphocyte ratio (PLR) have also been correlated to the inflammatory status of COVID-19 patients [9,10].

Albumin, as a negative acute-phase protein (APP), has previously been used as a prognostic biomarker in various infections, such as sepsis [12,13]. Indeed, its serum blood level decreases significantly with infection aggravation and reflects the severity of systemic inflammation [12,14]. Similarly, serum LDH levels tend to rise with increased infection severity and reflect the extent of infection-induced cellular injuries [15]. However, when used separately, both serum albumin and LDH levels can be affected by many medical conditions, aside from infection. For example, defective synthesis because of hepatocyte damage, excessive loss due glomerular diseases and deficient intake of amino acids due to malnutrition can cause sever hypoalbuminemia [12,16]. Similarly, liver disease, anemia, heart attacks and muscle traumas can elevate LDH serum levels [17,18,19].

As such, LDH to albumin ratio was introduced as new independent, composite prognostic factor for patients’ survival in severe infections, such as sepsis [20] but also in those with COVID-19 [21]. Nevertheless, the hunt for more such composite biomarkers is on. As abnormal kidney function [22] as well as blood urea nitrogen (BUN) to serum albumin ratio [23] were found to predict adverse outcomes in COVID-19 patients, the present study aims to assess the levels of yet another novel and more complex composite marker, namely Lactate dehydrogenase/Albumin to-urea ratio in hospitalised COVID-19 patients, with and without fatal clinical complications post infection.

## 2. Materials and Methods

### 2.1. Study Design and Participants

This is a retrospective cohort study that included patients with confirmed COVID-19 infection, hospitalised for acute complications between February 2020 and March 2021, at a single UK National Health Trust.

Patients were identified as COVID-19 positive by Reverse Transcriptase Polymerase Chain Reaction (RT-PCR) from throat/nose swabs on a ROCHE COBAS™ analyser (Roche Ltd., Basel, Switzerland). Nasopharyngeal or oropharyngeal samples were collected from patients for the detection of SARS-CoV-2 RNA. The Xpert^®^ Xpress SARS-CoV-2 (Cepheid Ltd., Sunnyvale, CA, USA) real-time RT-PCR assay was performed to achieve qualitative detection of SARS-CoV-2 RNA [9]. Ethical approvals were obtained through the Integrated Research Approval System (289571), sponsored by research and development committee of the Trust site (20Haem60) and was designed and conducted in accordance with the tenets of the Declaration of Helsinki [9].

**Figure 1:**
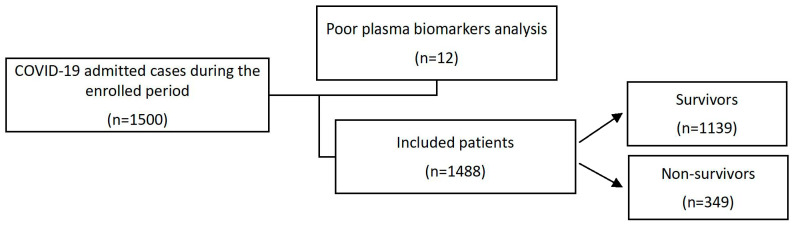
Flowchart of recruited and enrolled study participants.

Data from 1500 hospitalised patients were initially screened for inclusion in the study, of which 12 individuals were excluded based on the quality of their plasma biomarkers analysis. The remaining 1488 patients were included in the final analysis and classified into two groups according to their survival: group A, COVID-19 infection survivors (1139 patients), and group B, COVID-19 infection non-survivors (349 patients—Figure 1). Demographic information, clinical data and laboratory tests were collected from the patients’ hospital electronic medical records (EMR). All patients received treatment strategies that were recommended by the UK National Health Service (NHS) COVID-19 management protocols.

### 2.2. General Assessments

Standard anthropometric measures of height and weight were recorded to determine body mass index (BMI = weight/height). Systolic blood pressure (SBP), diastolic blood pressure (DBP), and heart rate (HR) were measured using an automatic Blood Pressure monitor (UA-767; A&D Instruments Ltd., Wokingham, UK) to determine mean arterial pressure (MAP = 2/3 DBP + 1/3 SBP) [24]. Eye opening and motor and verbal responses were assessed to all patients to objectively measure their level of consciousness using Glasgow Coma Score (GCS) [25].

### 2.3. Laboratory Procedures

Blood and plasma samples drawn from the antecubital fossa vein were assessed immediately for fasting glucose (GLUC), triglycerides (TG), total cholesterol (T-CHOL), high-density lipoprotein cholesterol (HDL-C), blood urea, bilirubin, alkaline phosphatase (ALP), alanine transaminase (ALT), aspartate aminotransferase (AST), creatinine (CRE) using the Reflotron Analyzer (Roche Diagnostics, Welwyn Garden City, UK). Low-density lipoprotein cholesterol (LDL-C) values were calculated using the Friedewald equation [26,27]. Lactate dehydrogenase (LDH), C-reactive protein (CRP) ANDferritin (FER) were examined using a clinical chemistry analyzer (Hitachi 7600; Sysmex, Kobe, Japan). Serum albumin levels (Alb) were measured using the ARCHI.TECT c Systems™ instrument (Abbot Laboratories, Diagnostic division Abbot Park, IL, USA) using the 7D53 BCG (Bromocresol Green) albumin assay kit (Abbot Laboratories, Diagnostic division Abbot Park, IL, USA).

A Sysmex™-XN (Sysmex Ltd., Tokyo, Japan) automated haematology analyser was used for complete blood count analysis including white blood cells (WBCs), haemoglobin (Hb), mean corpuscular volume (MCV), platelets (PLT), neutrophils (Neut), lymphocytes (Lymph), monocytes (Mono), eosinophils (Eos) and basophils (Baso) count. LAU ratio was calculated by dividing the LDH concentration by the albumin/urea concentration.

INR and D-Dimer values were measured using ACL TOP^®^ coagulation analyzer (Instrumentation Laboratory, Bedford, MA, USA). For D-Dimer a Latex Reagent was used, which is a suspension of polystyrene latex particles of uniform size coated with the F(ab’)2 fragment of a monoclonal antibody highly specific for the D-Dimer domain included in fibrin soluble derivatives to allow a more specific D-Dimer detection avoiding the interference of endogenous factors like the Rheumatoid Factor. When plasma, which contains D-Dimer, is mixed with the Latex Reagent and the Reaction Buffer included in the D-Dimer HS 500 kit (Instrumentation Laboratory, Bedford, MA, USA), the coated latex particles agglutinate. The degree of agglutination is directly proportional to the concentration of D-Dimer in the sample and is determined by measuring the decrease of the transmitted light caused by the aggregates (turbidimetric immunoassay).

For prothrombin time (PT) the principle of Coagulometric (turbidimetric) clot detection is used in the system to measure and record the amount of time required for a plasma specimen to clot. This technique assesses coagulation endpoint by measuring change in optical density.

INR is calculated using the following equation, where ISI is the international sensitivity index. All laboratory tests were conducted within 3 days of COVID-19 diagnosis.

$$\mathrm{INR}={\left(\frac{\mathrm{PT}\;\mathrm{test}}{\mathrm{PT}\;\mathrm{notmal}}\right)}^{\mathrm{ISI}}$$

### 2.4. Sample Size and Statistical Analysis

As the study design was multifactorial in nature, it was calculated that a sample size of *n* = 1488 is sufficient to provide 80% power at an alpha level of 0.05. All analyses were performed using SPSS^®^ statistical software (version 25, IBM Corp., Armonk, NY, USA). Distributions of continuous variables were determined by the Shapiro–Wilk test. In cases where the normality of the data could not be confirmed, appropriate data transformations were made, or non-parametric statistical alternatives were used. Univariate associations were determined using Pearson’s (normally distributed data) or Spearman’s method (non-normally distributed data), and forward stepwise regression analyses were performed to test the influence of measured clinical outcomes and the circulatory biomarkers [9]. Differences between groups were subsequently assessed using independent-samples *t*-test or ANCOVA, as appropriate. *p* < 0.05 was considered statistically significant. A receiver operating characteristic curve analysis was used to assess the diagnostic ability and accuracy of LAU ratio [9].

## 3. Results

### 3.1. Patient Characteristics

Patients’ characteristics are described in Table 1. There were statistically significant differences between the 2 study groups with regard to mean age (*p* = 0.0001) and respiratory rate (*p* < 0.001). No statistically significant differences were found between the two groups with regard to SBP, DBP, MAP, HR, BMI and GCS.

### 3.2. Haematology Results

Compared to the survivors’ group, the COVID-19 non-survivors’ group exhibited significantly lower levels of Hb and PLT (*p* > 0.005), as well as a higher mean MCV and Neut count (*p* = 0.001 and *p* = 0.0013, respectively). Similarly, NLR and LAU ratios were significantly higher in the non-survivors’ group (all *p* < 0.0001, Table 2).

### 3.3. Organ Function Tests

COVID-19 non-survivors’ group showed statistically significant higher international normalized ratio (INR) (*p* = 0.003), D-dimer (*p* = 0.029), blood urea (*p* < 0.0001), total bilirubin (*p* < 0.005), ALP (*p* = 0.003), serum CRE (<0.0001), CRP (<0.0001), FER (*p* = 0.021) and LDH (*p* = 0.032), as well as lower serum albumin levels (*p* < 0.0001) than the COVID-19 survivors’ group (Table 3).

### 3.4. Correlation Studies

**Figure 2:**
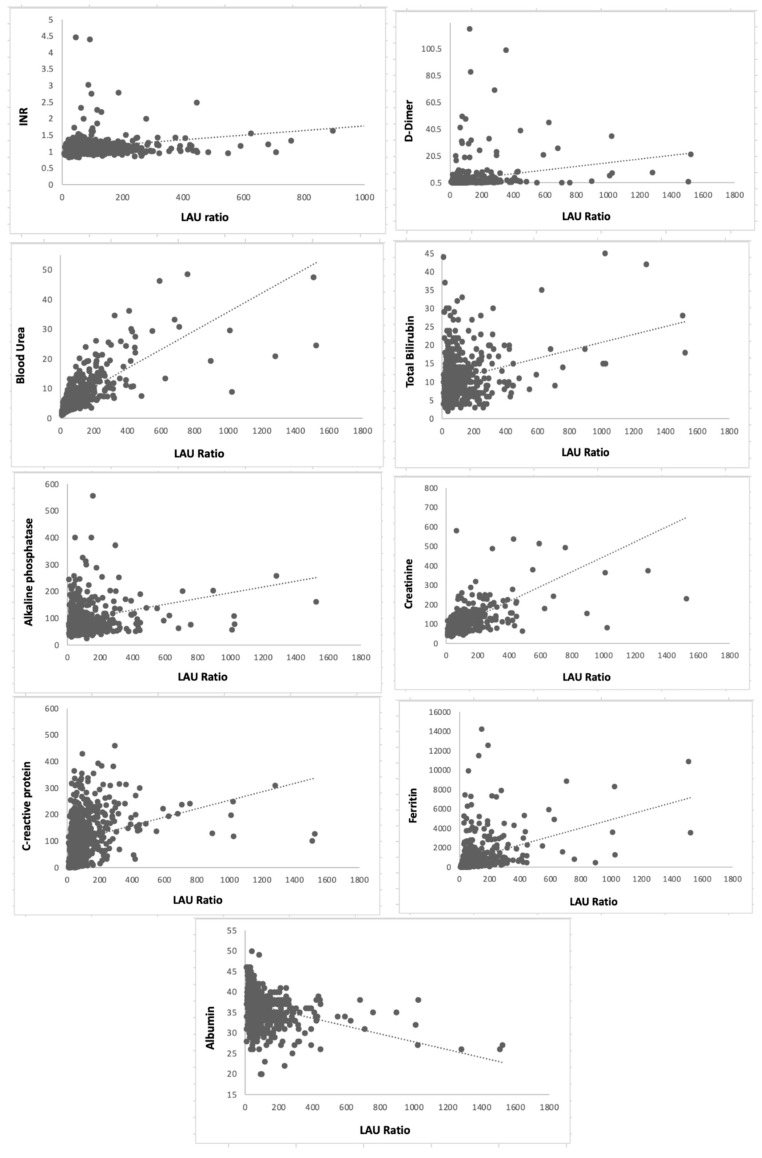
Correlation studies between Lactate dehydrogenase/Albumin to-urea ratio (LAU) and Infection-related clinical complications prognostic parameters.

There were significantly positive correlations between LAU ratio and INR (r = 0.171), D-dimer (r = 0.176), blood urea (r = 0.424), serum total bilirubin (r = 0.107), ALP (r = 0.115), CRE (r = 0.365), CRP (r = 0.268) and FER (r = 0.385) and negatively with ALB (r = −0.114), (all *p* < 0.0001 in all) (Figure 2).

### 3.5. Analysis of the Area under Receiver Operating Characteristic (AUROC)

On comparison between the two ratios the LAU ratio had an area under receiver operating characteristic (AUROC) of 0.67 (95% confidence interval 0.56 to 0.79) compared to 0.60 (95% confidence interval 0.54 to 0.68) using NLR. The ideal LAU ratio cut-off for in-hospital mortality using Youden’s index was 54.7.

## 4. Discussion

COVID-19 remains a major health- related burden worldwide with a global mortality estimate of 14.9 million deaths [28]. Over the pandemic period it was identified that the most critical step in the management of COVID infection is the quick identification of high-risk patients before the rapid deterioration to critical conditions that involve end-organ dysfunction [29]. As a result, numerous attempts were made to identify a clinically valid, cost-effective biomarker for risk stratification and prognostication of infected patients to provide optimal management as soon as possible [30]. During these attempts numerous risk factors were suggested as stratification markers of mortality [31]. Of them, older age, chronic comorbidities such as chronic obstructive pulmonary diseases, renal failure, cardiovascular diseases, and obesity were linked to virus-induced clinical complications.

Various studies have also addressed inflammation induced neutrophils production stimulation and lymphocytes apoptosis and consequently high NLR as the gold standard marker of severe COVID-19 infection [32]. However, the dysregulation of body immune responses due to the infection was found to result in abnormal haematological reactions and thus clinically misleading NLR values [33,34]. Additionally, many studies have also reported that leukocytosis is only observed in patients who deteriorate clinically and not in the mid and moderate cases [32,33,34,35,36]. Thus, differences in biomedical markers were suggested as an improved prognostic marker of COVID-19 induced critical illness and death.

Similar to severe bacterial illness, it was found that the aggravation of viral load induces abnormalities in several blood biomarkers such as cytokines, APPs and organ dysfunction-related markers [37,38]. In line with other studies, our study showed that serum blood levels of many of these biomarkers such as CRP, blood urea, total bilirubin, CRE, ALP, albumin and LDH increase significantly with infection exacerbation [13,39]. Our results also supported this phenomenon with refractory patients exhibiting abnormal liver function tests presented as high serum bilirubin, ALT, ALP; kidney dysfunction presented as elevated blood urea, high creatinine levels, and abnormal myocardial zymograms shown as elevated CK levels.

LDH is a cellular energy marker present in all living cells. Thus, any cell damage owing to infection, organ injury or systemic inflammation leads to increased serum LDH concentration [13]. Consequently, it has been used as a prognostic factor for many infections and pathologies inducing cell injuries such as malignancies, haemolysis, shock, and inflammatory disorders [15,40,41]. Our study revealed significantly high LDH serum levels in the non-survival group compared to the COVID-19 survival patients. Furthermore, and unlike LDH, serum albumin concentration as a negative APP representative of inflammation was lower in the non-survival group compared to the COVID-19 survivals. This is consistent with findings from other studies highlighting hypoalbuminemia as a strong predictor of poor clinical outcomes [42].

In comparison to LAU, NLR failed to show the same strong correlation with multiple end-organs dysfunction biomarkers.

While NLR failed to show this, our data also showed that male patients had higher LAU ratio in both groups with a more significant correlation to mortality risk factors than the female patients which supports results from other studies showing that male patients have higher rates of COVID-19 refractoriness [43].

Finally, there are some limitations that should be noted in this study. First, although we adjusted our results for the main cofounders, still residual cofounding variables might have effect in our results. Second, the number of organ dysfunction evaluation tests are limited which might affect the statistical power of the study. Testing these results in different ethnic subgroups is also recommended by the authors to ensure validity and reliability.

As discussed above, ALB and LDH has been usually used separately to assess severity, however no study assessed their prognostic efficacy as a ratio in infectious diseases. In our study we showed for the first time that the LAU ratio can be used independently to stratify high risk COVID-19 patients. Our study also showed that compared to the COVID-19 prognostic gold standard NLR, LAU was more potent in the premature identification of severe illness risk factors even in the mild and moderate cases. We believe the results of this study have important clinical implications since LAU can be quickly measured on emergency blood tests upon admission. This may facilitate appropriate medical treatment such as oxygen and antiviral drugs with prompt access to the intensive care unit at an early stage, if necessary. Consequently, this can reduce in-hospital mortality and alleviate medical management for COVID-19 patients.

The authors recommend routine evaluation of LAU ration in all COVID-19 patients and to consider a value above 54.7 as a positive indicator of infection-related organ dysfunction. We also recommend early hospitalization, intensive monitoring, and prompt clinical interventions to reduce infection related morbidity and mortality in these patients. More research is needed to establish the external validity of our results.

## 5. Conclusions

Patients with a high LAU ratio are at increased risk of mortality due to COVID-19 infection. Therefore, early assessment of this parameter and intensive intervention could improve their prognosis.

## Figures and Tables

**Table 1:** Basic Characteristics of the Study Population.

	GP (A) Survivors (1139)	GP (B) Non-Survivors (349)	*p*-Value
Age (year)	58.66 (18.90)	75.87 (13.43)	0.0001 *
RR (12–18 bpm)	19.24 (5.53)	21.05 (6.24)	<0.001 *
SBP (mmHg)	121 (13.78)	126.4 (14.3)	0.76
DBP (mmHg)	73.01 (16.199)	71.08 (14.15)	0.06
MAP (mmHg)	91 (12.45)	92.4 (11.37)	0.37
HR (60–100 bpm)	81.84 (20.68)	83.50 (21.03)	0.19
BMI (18.5–24.9 Kg/m^2^)	22.5 (4.6)	23.1 (5.7)	0.23
GCS	9.60 (7.0)	10.20 (6.23)	0.16

Abbreviations: RR, respiratory rate; bpm, breath per minute; SBP, systolic blood pressure; mmHg, millimeter of mercury; DBP, diastolic blood pressure; MAP, mean arterial blood pressure; HR, heart rate; bpm, beat per minute; BMI, body mass index; GCS, Glasgow Coma Score. * Significant *p*-values are indicated where *p* < 0.05 was considered significant.

**Table 2:** Haematological Findings of the Study Population.

	GP (A) Survivors (1139)	GP (B) Non-Survivors (349)	*p*-Value
HB-A1c (<42 mmol/mol)	52.22 (19.46)	50.75 (16.38)	0.530
T-CHOL (<5 mmol/L)	4.88 (3.48)	4.39 (0.99)	0.329
WBCs (4–11 × 10^9^/L)	8.004 (7.33)	9.00 (10.1)	0.095
Hb (115–160 g/L)	131.72 (21.47)	122.81 (24.52)	0.0001 *
MCV (80–100 fL)	86.08 (7.82)	90.01 (8.80)	0.0001 *
PLT (150–450 × 10^9^/L)	255.85 (106.17)	232.49 (104.83)	0.0001
Neut (1.7–7.5 × 10^9^/L)	5.92 (4.47)	6.82 (4.37)	0.0013 *
Lymph (1–4 × 10^9^/L)	1.32 (1.042)	1.51 (8.58)	0.695
Mono (0.2 * 80 × 10^9^/L)	0.6161 (1.722)	0.596 (0.44)	0.839
Eos (>0.5 × 10^9^/L)	0.058 (0.13)	0.059 (0.30)	0.877
Baso (>0.1 × 10^9^/L)	0.024 (0.023)	0.025 (0.032)	0.859
Neut/Lymph	6.173 (6.095)	9.52 (9.66)	<0.0001 *
LDH/ALB X Urea	36.51 (92.64)	81.63 (168.70)	<0.0001 *

Abbreviations: HB-A1c, haemoglobin A1C; T-CHOL, total cholesterol; WBCs, white blood cells; Hb, haemoglobin; MCV, mean corpuscular volume; PLT, platelets; Neut, neutrophils; Lymph, lymphocytes; Mono; monocytes; Eos, eosinophils; Baso, basophils; Neut/Lymph, neutrophils to lymphocytes ratio; LDH/ALB X Urea, Lactate dehydrogenase/Albumin to-urea ratio. * Significant *p*-values are indicated where *p* < 0.05 was considered significant.

**Table 3:** Infection-related Clinical Complications Prognostic Parameters.

	GP (A) Survivors (1139)	GP (B) Non-Survivors (349)	*p*-Value
INR (0.9–1.2)	1.12 (0.29)	1.25 (0.69)	0.003 *
D-dimer (0.22–0.46 FEU µg/mL)	2.96 (9.48)	4.98 (11.92)	0.029 *
Urea (2.5–7.8 mmol/L)	6.90 (5.70)	11.43 (7.74)	0.0001 *
Albumin (35–50 g/L)	38.37 (11.66)	33.52 (4.92)	0.0001 *
Bilirubin (<21 µmol/L)	10.56 (10.98)	13.11 (15.30)	0.005 *
ALP (30–130 IU/L)	95.13 (59.73)	117.13 (128.61)	0.003 *
ALT (4–36 U/L)	40.77 (48.69)	43.90 (80.85)	0.510
CRE (45–110 µmol/L)	102.43 (9795)	130.91 (77.09)	0.0001 *
CRP (<1 mg/L)	84.53 (82.43)	118.56 (91.51)	0.0001 *
FER (41–400 μg/L)	854.92 (1324.32)	1180.42 (1818.14)	0.021 *
LDH (140–280 U/L)	442.18 (259.71)	507.349 (341.38)	0.032 *
cTnI (<14 ng/L)	207.37 (2256.01)	219.96 (1038.96)	0.938
25-OHD (25–40 ng/mL)	43.53 (31.05)	46.65 (41.97)	0.253

Abbreviations: INR, international normalized ratio; ALP, alkaline phosphatase; ALT, Alanine transaminase; CRE, creatinine; CRP, C-reactive protein; FER, ferritin; LDH, lactate dehydrogenase; cTnI, cardiac troponin-I; 25OHD, 25-hydroxycholecalciferol. * Significant *p*-values are indicated where *p* < 0.05 was considered significant.

## Data Availability

Data supporting this research cannot be shared due to ethical considerations (patient medical records).
